# Chromosome architecture and homologous recombination in meiosis

**DOI:** 10.3389/fcell.2022.1097446

**Published:** 2023-01-06

**Authors:** Masaru Ito, Akira Shinohara

**Affiliations:** Institute for Protein Research, Osaka University, Suita, Osaka, Japan

**Keywords:** cohesin, axis-loop structure, synaptonemal complex, meiotic recombination, crossover

## Abstract

Meiocytes organize higher-order chromosome structures comprising arrays of chromatin loops organized at their bases by linear axes. As meiotic prophase progresses, the axes of homologous chromosomes align and synapse along their lengths to form ladder-like structures called synaptonemal complexes (SCs). The entire process of meiotic recombination, from initiation *via* programmed DNA double-strand breaks (DSBs) to completion of DSB repair with crossover or non-crossover outcomes, occurs in the context of chromosome axes and SCs. These meiosis-specific chromosome structures provide specialized environments for the regulation of DSB formation and crossing over. In this review, we summarize insights into the importance of chromosome architecture in the regulation of meiotic recombination, focusing on cohesin-mediated axis formation, DSB regulation *via* tethered loop-axis complexes, inter-homolog template bias facilitated by axial proteins, and crossover regulation in the context of the SCs. We also discuss emerging evidence that the SUMO and the ubiquitin-proteasome system function in the organization of chromosome structure and regulation of meiotic recombination.

## Introduction

**FIGURE 1:**
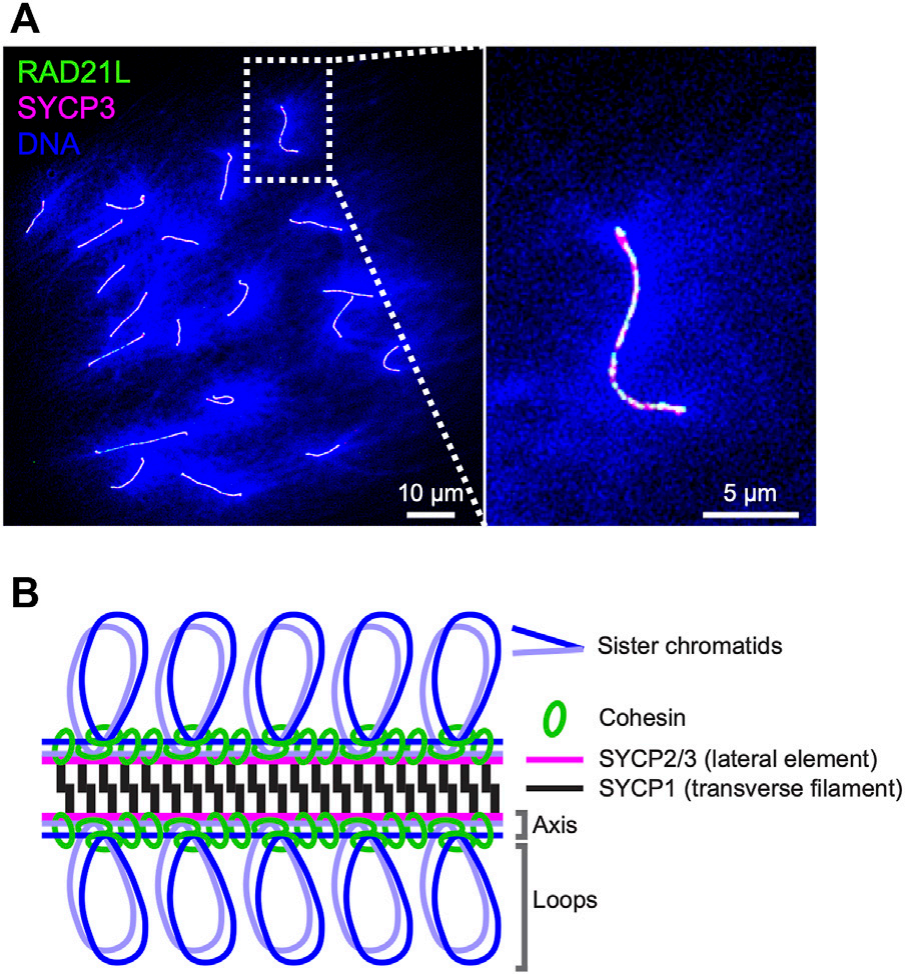
Axis-loop chromosome structure and the synaptonemal complex in mice **(A)** Surface spreads of mouse oocyte pachytene chromosomes immunostained for RAD21L (green), SYCP3 (magenta), and DNA (DAPI; blue). DNA is condensed on chromosome axes where cohesin complexes and axis core proteins localize and spread as loops from axes. RAD21L and SYCP3 are shown as a representative of meiotic cohesin and axis core protein, respectively **(B)** Schematic representation of the mouse synaptonemal complex. Cohesin complexes interconnect axes of sister chromatids and lateral elements SYCP2 and SYCP3 and a transverse filament protein SYCP1 form a ladder/zipper-like structure.

Homologous recombination during meiosis underlies biological diversity and ensures proper chromosome segregation during the first division to create haploid gametes. During meiotic prophase-I, chromosomes develop highly organized three-dimensional structures where loops of chromatin emanate from structural axes that also interconnect sister chromatids. Programmed DNA double-strand breaks (DSBs) at recombination hotspots, which initiate meiotic recombination, are localized to DNA sequences found in chromatin loops while many factors responsible for DSB formation reside on the axes, indicating that tethering of DSB sites in loops to their corresponding chromosome axes–loop-axis tethering–is a crucial step in the initiation of meiotic recombination (Blat et al., 2002; Panizza et al., 2011). Following DSB formation, homolog search of the DSB ends for homologous chromosomes leads to pairing of the structural axes of two homologous chromosomes and synapsis along their lengths to form the synaptonemal complexes (SCs). The SCs are zipper-like structures where the lateral/axial elements localized to each homolog sandwich the central region composed of transverse filaments and a central element (Figure 1). Later steps of recombination such as the formation of double-Holliday junctions and their resolution into crossover products, occur within the context of the SCs. In this review, we present key findings about the regulation of meiotic recombination in relation to chromosome architecture.

## Cohesin as a basis of axis-loop higher-order chromosome structure

During the meiotic S-phase, cohesin complexes interconnect sister chromatids and are assumed to establish the core unit of chromosome axis *via* loop extrusion, likely with help of evolutionarily related axis core proteins (budding yeast Red1, mammalian SYCP2/SYCP3, and plant ASY3/ASY4; West et al., 2019; Figures 1A,B). The cohesin complexes consist of two SMCs (structure maintenance of chromosome), SMC1 and SMC3; and two non-SMC kleisin subunits, SCC3/STAG and the α-kleisin RAD21/SCC1 (Nasmyth and Haering, 2005). REC8 is a meiosis-specific α-kleisin subunit that is well-conserved from yeast to mammals and is required for the formation of chromosome axes and the SCs in budding yeast, *C. elegans*, and mice (Klein et al., 1999; Pasierbek et al., 2001; Xu et al., 2005). Recent Hi-C analysis of yeast meiosis revealed Rec8-dependent intra-chromosome interactions between distal chromosomal loci and high-frequency contacts between Rec8 binding sites (Muller et al., 2018; Schalbetter et al., 2019), supporting a model in which interactions between adjacent cohesin-binding sites assemble structural axes. Deletion of the budding yeast *REC8* gene causes various defects in meiotic recombination; the redistribution and reduction of DSBs, impaired choice of recombination template, and persistence of joint molecule DNA intermediates (Kugou et al., 2009; Kim et al., 2010), indicating important roles of cohesin-mediated chromosome structures and/or the cohesin complexes themselves in the regulation of recombination. Recent work in fission yeast identified a *rec8* separation-of-function mutant, *rec8-F204S,* that is proficient for sister chromatid cohesion (SCC) but deficient for axis-loop structure (Sakuno et al., 2022). This *rec8* mutant was defective in meiotic recombination, revealing an essential role for Rec8-cohesin-mediated axis-loop chromosome structure and not cohesion *per se* in meiotic recombination.

In mice, the topologically associating domains (TADs, comprising ∼1 Mbp-intra-chromosomal interactions), characteristic of interphase chromosomes, are diminished and intra-chromosomal interactions around 2.5–4.5 Mbp became more evident during meiotic prophase-I, consistent with the formation of axis-loop structures (Alavattam et al., 2019; Wang Y. et al., 2019; Patel et al., 2019; Vara et al., 2019; Luo et al., 2020; Zuo et al., 2021). REC8, SMC1β, STAG3, and RAD21L (a second meiosis-specific α-kleisin; Figure 1A) are known meiosis-specific cohesin subunits that localize to chromosome axes in mice as six distinct complexes; three SMC1β-cohesin complexes (RAD21-SMC1β-SMC3-STAG3, RAD21L-SMC1β-SMC3-STAG3, and REC8-SMC1β-SMC3-STAG3) and three SMC1α-cohesin complexes (RAD21-SMC1α-SMC3-STAG1/2, RAD21-SMC1α-SMC3-STAG3, and RAD21L-SMC1α-SMC3-STAG3) (Revenkova et al., 2004; Ishiguro et al., 2011; Lee and Hirano, 2011; Fukuda et al., 2014). With the exception of *Rad21L* ^*−/−*^ females, all mice that are knockout mutants for the meiosis-specific cohesin components are sterile, and show defects in synapsis and compromised meiotic recombination (Revenkova et al., 2004; Herran et al., 2011; Llano et al., 2012; Fukuda et al., 2014). Axis lengths in meiocytes are shorter in all mutants, and double mutant mice such as *Smc1β* ^*−/−*^ *Rec8* ^*−/−*^ show much shorter axis lengths than the corresponding single mutants (Biswas et al., 2016; Ward et al., 2016). These observations highlight the importance of the multiple cohesin complexes in the organization of meiotic chromosome axis structure in mice.

During meiosis, cohesin plays a dual role in sister chromatid cohesion (SCC) and the formation of axis-loop structure. A recent series of studies established the loop extrusion activity of SMC complexes including the mitotic SCC1/RAD21-based cohesin (RAD21-SMC1A-SMC3-STAG1), which requires the cohesin loader complex SCC2/NIPBL-SCC4/MAU2 (Davidson et al., 2019; Kim et al., 2019; Kaur et al., 2022). This loop extrusion seems to be distinct from cohesin’s SCC activity (Davidson and Peters, 2021). It follows that meiotic chromosome structure and cohesion may be mediated by two independent ensembles of cohesin complexes. Importantly, several organisms including vertebrates and nematodes contain two distinct meiotic cohesins (Severson et al., 2009; Ishiguro et al., 2011; Lee and Hirano, 2011; Severson and Meyer, 2014). In mice, the cohesins with REC8 and RAD21L localize to non-overlapping sites along chromosome axes (Ishiguro et al., 2011). Moreover, REC8, and thus REC8-based cohesin, localizes to the chromosomes as early as meiotic S-phase and persists until metaphase-II; whereas RAD21L-cohesin appears on the chromosome later, in leptonema and disappears earlier in late prophase-I (Herran et al., 2011; Lee and Hirano, 2011; Ishiguro et al., 2014; Biswas et al., 2016). One simple idea is that REC8-cohesin functions for SCC and RAD21L-cohesin functions for loop extrusion and thus axis-loop formation. Future studies are essential to evaluate the hypothesis.

WAPL and PDS5 are highly conserved cohesin regulators that contribute to the association and dissociation of cohesin complexes from chromosomes, and thereby modulate chromosome architecture in somatic cells (Kueng et al., 2006; Tedeschi et al., 2013; Haarhuis et al., 2017; Wutz et al., 2017). In *C. elegans*, cytological analysis of *wapl-1* null mutants indicated minor defects in the repair of meiotic DSBs (Crawley et al., 2016). Physical analysis of meiotic recombination at a well-characterized DSB hotspot in budding yeast revealed a subtle reduction in the levels of meiotic DSBs and the homolog bias of DSB repair in *rad61/wpl1* deletion mutants (Challa et al., 2016; Hong et al., 2019). More severe defects were seen in *pds5* meiotic null mutants with an interhomolog bias defect similar to that of a *rec8* deletion mutant (Hong et al., 2019). Both *rad61/wpl1* and *pds5* mutants showed shortened chromosome axes in budding yeast (Challa et al., 2016; Yang et al., 2022) and in fission yeast (Ding et al., 2006; Sakuno et al., 2022). Importantly, the budding yeast *pds5* mutant forms SCs between sister chromatids instead of homologs (Jin et al., 2009), which is reminiscent of the phenotypes seen in mouse *Rec8, Rad21L*, *Stag3*, and *Smc1β* knockout mutant spermatocytes (Xu et al., 2005; Ishiguro et al., 2011; Llano et al., 2012; Agostinho et al., 2016). Recent studies also revealed that depletion of PDS5 (both PDS5A and PDS5B) in mice leads to shortened chromosome axes, which form normal SCs between homologs, but are compromised for meiotic recombination (Viera et al., 2020). The prophase-I phenotypes of *Wapl* mutant mice have not been reported yet.

Notably, budding yeast Pds5 interacts with the proteasome and the shortened chromosome axis length of *pds5* mutants is rescued by reducing levels of ubiquitin, suggesting that Pds5 regulates axis length *via* the ubiquitin-proteasome system (Yang et al., 2022). Consistently, the proteasome is indeed localized on chromosome axes in budding yeast, *C. elegans*, and mice (Ahuja et al., 2017; Rao et al., 2017). Although changes in chromosome structures resulting from mutation of *PDS5* might indirectly affect meiotic recombination in mice, physical interaction between PDS5 and two RAD51 mediators, BRCA2 and the SWS1-SWSAP1, has been reported. Moreover, DSB repair is defective in *PDS5* mutant somatic cells from fly and human (Brough et al., 2012; Kusch, 2015; Couturier et al., 2016; Martino et al., 2019). These data support more direct roles for PDS5 in meiotic recombination, either as a component of cohesin or as an independent complex.

## DSB formation in tethered loop-axis complexes

**FIGURE 2:**
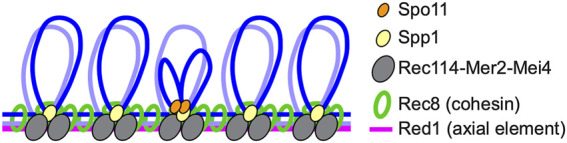
Tethered loop-axis complex (TLAC) formation to regulate DSB formation in budding yeast. Schematic representation of the budding yeast TLAC. Spo11 partner Rec114-Mer2-Mei4 complex localizes to chromosome axes where Rec8 cohesin and an axial element Red1 reside, and Spp1, a component of the Set1/COMPASS complex, tethers Spo11-bound DSB hotspots within loops to chromosome axes *via* the interaction with Mer2.

Meiotic recombination is initiated by programmed DSBs formed *via* an evolutionarily conserved topoisomerase VI-like protein, Spo11, and its partners (Bergerat et al., 1997; Keeney et al., 1997; de Massy, 2013; Robert et al., 2016). DSB sites are located in chromatin loops while Spo11 partners such as Rec114-Mer2-Mei4 in budding yeast, Rec7-Rec15-Rec24 in fission yeast, and REC114-IHO1-MEI4 in mice localize to chromosome axes where cohesin also localizes, suggesting that tethered loop-axis complexes (TLACs) form during the initiation of meiotic recombination to regulate both DSB formation and the ensuing steps of meiotic recombination (Blat et al., 2002; Kumar et al., 2010; Panizza et al., 2011; Miyoshi et al., 2012; Fowler et al., 2013; Ito et al., 2014; Kumar et al., 2015; Stanzione et al., 2016); (Figure 2).

Molecular mechanisms of TLAC formation have been studied in yeasts. Spp1 in budding yeast and Mde2 in fission yeast are identified as proteins important for the formation of TLACs (Miyoshi et al., 2012; Acquaviva et al., 2013; Sommermeyer et al., 2013; Adam et al., 2018). In budding yeast, DSB hotspots are preferentially located in promoter regions within chromatin loops (Pan et al., 2011; Ito et al., 2014). Spp1, a component of the COMPASS/Set1 complex that catalyzes histone H3K4 trimethylation, is thought to recognize H3K4 trimethylation marks around DSB hotspots *via* its PHD domain, and connect these sites to chromosome axes by interacting with axis-associated Mer2. Spp1 is likely to mediate TLAC formation independently from the role in the COMPASS/Set1 complex (Karanyi et al., 2018). Although Spp1-mediated TLACs contribute to DSB formation, meiotic cells are equipped with another layer of regulation for meiotic DSB formation, since *spp1* mutants still form relatively high levels of DSBs (Acquaviva et al., 2013; Sommermeyer et al., 2013; Zhang et al., 2020). Given that Mer2 itself has an ability to directly bind to nucleosomes and the association of Mer2 to chromosome axes is regulated by its interacting axis-associated protein Hop1, the Hop1-Mer2 may contribute to TLAC formation both *via* and independently of Spp1 (Panizza et al., 2011; Rousova et al., 2021). In fission yeast, where most DSB hotspots are in long intergenic regions (Fowler et al., 2014), DSB hotspots are marked by another epigenetic mark, H3K9 acetylation, and the H3K4 trimethylation mark is dispensable for meiotic DSB formation (Yamada et al., 2013). Mde2 expresses only after the meiotic S-phase and is thought to bridge Rec12^Spo11^-containing subcomplex at DSB hotspots and an axis-located subcomplex containing Rec15^Mer2^ (budding yeast homologs in superscript) (Miyoshi et al., 2012). Importantly, fission yeast Hop1 also physically interacts with Rec15^Mer2^ and promotes chromosomal localization of Rec15^Mer2^, suggesting significant contribution of Hop1 to TLAC formation in both yeasts (Kariyazono et al., 2019).

Whether or not the mechanism of TLAC formation is conserved remains unclear. In mice, PRDM9, a germ cell-specific H3K4 trimethylation transferase with a zinc-finger array domain, recognizes specific DNA sequences, deposits H3K4me3 and H3K36me3 marks, and directs DSB formation at its binding sites (Baudat et al., 2010; Diagouraga et al., 2018). Recent ChIP-seq analysis for meiotic cohesin components REC8 and RAD21L revealed their localization to promoter regions (Vara et al., 2019) and no overlap of meiotic cohesin binding sites with DMC1 (the meiosis-specific RAD51 homolog) and PRDM9 binding sites (Jin et al., 2021). CXXC1 is an ortholog of budding yeast Spp1, and the physical interaction of CXXC1 with PRDM9 and IHO1, an axis-associated protein considered to be the ortholog of budding yeast Mer2, suggested a similar mechanism of TLAC formation between budding yeast and mouse (Imai et al., 2017; Parvanov et al., 2017). However, depletion of CXXC1 in mouse germ cells caused no or small defects in DSB formation and the early steps of DSB repair (Tian et al., 2018; Jiang et al., 2020), suggesting that factor(s) other than CXXC1 plays a critical role in TLAC formation and meiotic DSB formation in mice. A mammalian ortholog of fission yeast Mde2 has not been identified yet.

## Inter-homolog bias controlled by axial proteins

DSB formation is followed by nuclease-mediated 5′-strand resection to form long single-stranded tails. Invasion of the resected DSB end into a template homologous duplex DNA forms a nascent D (displacement)-loop structure. At this stage, D-loop intermediates are thought to differentiate into crossover and non-crossover pathways (Hunter, 2015). The majority are matured as non-crossovers *via* DNA synthesis to extend the invading end, dissociation of the D-loop, and annealing of the displaced strand to seal the DSB (synthesis-dependent strand annealing) (Allers and Lichten, 2001; Hunter and Kleckner, 2001). Along the crossover pathways, D-loops differentiate into metastable D-loops called Single-End Invasions (SEIs) which then form double-Holliday junctions (dHJs) *via* DNA synthesis and capture of the second DSB end. dHJs are specifically resolved into crossover products. These events also occur in the context of meiotic chromosome axes and SCs. A prominent feature of meiotic recombination is that homology search and strand exchange are biased to occur between homologous chromosomes (inter-homolog) rather than between sister chromatids (inter-sister). This biased template choice is regulated by components of the axial/lateral elements of the SC and axis-associated proteins.

In budding yeast deletion mutants of axis-associated proteins Red1, Hop1, and the associated recombination-checkpoint kinase Mek1, DSBs are repaired primarily *via* inter-sister recombination (Kim et al., 2010; Lao and Hunter, 2010). The Hop1-Red1-Mek1 pathway, along with other factors that promote inter-homolog recombination (Zierhut et al., 2004), may mediate inter-homolog bias by inhibiting inter-sister recombination, promoting inter-homolog recombination, and/or by impeding the progression of recombination until homologs have been engaged (Lao and Hunter, 2010). Further mutant analysis suggested that meiotic cohesin Rec8 promotes inter-sister bias, which is counteracted by Red1 and Mek1/Mre4 (Kim et al., 2010). Mek1 is a meiosis-specific, axis-associated kinase that phosphorylates various targets including Rad54 and Hed1. The phosphorylation of both Rad54 and Hed1 suppresses Rad51-mediated inter-sister recombination, which partly explains the involvement of Mek1 in the suppression of inter-sister recombination (Niu et al., 2007; Niu et al., 2009; Callender et al., 2016; Kniewel et al., 2017). Importantly, the meiotic Rad51 homolog, Dmc1, bears an ability to promote inter-homolog bias (Brown and Bishop, 2014). However, the exact mechanism of inter-homolog bias and the relationship between Mek1-mediated phosphorylation and Rec8-cohesin remain to be resolved.

Hop1 is a conserved HORMA domain-containing protein that specifically localizes to unsynapsed axes and is locally depleted from sites of synapsis (Smith and Roeder, 1997), distinct from its binding partner Red1 and the cohesin complexes that appear to be constitutive components of chromosome axes before and after SC formation. Removal of Hop1 from synapsed axes is mediated by an evolutionarily conserved AAA+ ATPase Pch2, and yeast *pch2Δ* mutants show increased inter-sister recombination, suggesting that Pch2 also contributes to inter-homolog bias *via* the Hop1-Red1-Mek1 axis (Borner et al., 2008; Zanders et al., 2011). In mice, the two HORMA domain-containing proteins HORMAD1 and HORMAD2 also preferentially localize to unsynapsed axes (Wojtasz et al., 2009). In the absence of the HORMADs, the repair of radiation-induced exogenous DSBs was accelerated in *Spo11*-and *Dmc1*-deficient meiocytes in which inter-sister recombination is preferred, suggesting that, like budding yeast Hop1, mouse HORMADs may impede inter-sister recombination (Shin et al., 2013; Rinaldi et al., 2017; Carofiglio et al., 2018). The removal of HORMADs from synapsed axes is mediated by the Pch2 homolog TRIP13 (Wojtasz et al., 2009; Roig et al., 2010; Ye et al., 2017). In *Trip13* mutant meiocytes, unrepaired DSBs persist (Li and Schimenti, 2007; Roig et al., 2010; Rinaldi et al., 2017), supporting the idea that HORMADs suppress inter-sister DSB repair.

## Synaptonemal complexes and crossing over

Synaptonemal Complexes (SCs) are tripartite protein structures where the two lateral/axial elements of homologous chromosomes are connected along their lengths by a central region comprising tightly-packed transverse filaments and a central element. The dependency of SC formation on DSBs and recombination differs among species, with recombination-dependent synapsis in most analyzed fungi, plants, and mammals where SC formation tends to initiate at sites of recombination (SC also initiates at centromeres in budding yeast). By contrast, DSBs are dispensable for the SC formation in *Drosophila* and *C. elegans* in which synapsis initiates at centromeres and terminal pairing centers, respectively (MacQueen et al., 2005; Takeo et al., 2011). Despite these differences, SCs have a common function in the formation and/or regulation of crossing over in all organisms (with known exceptions being *Schizosaccharomyces pombe* and *Aspergillus nidulans* that have no typical SC structure).

The ZMM proteins are a group of meiosis-specific proteins that facilitate crossing over by promoting/stabilizing the crossover-pathway joint-molecule intermediates, SEIs and dHJs, and promoting the crossover-specific resolution of dHJs *via* MutLγ. Initially identified in budding yeast, the ZMMs comprise eight members that define five structures or activities: Zip1^SYCP1^ is the transverse filament components of SCs but also functions locally at recombination sites; Zip2^SHOC1^-Spo16 is related to XPF-ERCC1 and thought to bind and stabilize recombination intermediates; Zip4^TEX11^ is a long TPR-repeat protein that appears to bridge chromosome axes and recombination complexes by forming the ZZS complex with Zip2^SHOC1^-Spo16; Zip3^RNF212^ is inferred to be an E3-ligase for SUMO modification that promotes the localization of other ZMMs to recombination sites; Msh4-Msh5 (MutSγ), homologous to DNA mismatch-repair factor MutS, binds and stabilizes joint molecules; and Mer3^HFM1^ is a DNA helicase that stabilizes joint molecules and regulates the length of recombination-associated DNA synthesis (mammalian homologs in superscript) (Lynn et al., 2007; De Muyt et al., 2018; Arora and Corbett, 2019). In budding yeast, all ZMM proteins are also required for SC formation, with Zip2-Spo16-Zip4 and Zip3 being defined as synapsis initiation complexes (SICs) that assemble at synapsis initiation sites, which mature into crossover sites, indicating a close link between SC initiation and crossing over at least in budding yeast and similarly in *Sordaria macrospora* (Chua and Roeder, 1998; Agarwal and Roeder, 2000; Borner et al., 2004; Fung et al., 2004; Tsubouchi et al., 2006; Shinohara et al., 2008; Zhang et al., 2014a). In mice, the number of ZMM-associated recombination sites, detected as cytological foci, is in large excess relative to SC-initiation sites and crossovers. Meiocytes from mouse *zmm* knockouts for *Hfm1, Msh4*, *Msh5*, *Shoc1*, *Spo16,* and *Tex11* show defects in synapsis and crossover formation, as seen in budding yeast. The exception is mouse knockout mutant for the *ZIP3* homolog *Rnf212*, in which synapsis occurs efficiently but crossing over fails (de Vries et al., 1999; Kneitz et al., 2000; Yang et al., 2008; Guiraldelli et al., 2013; Reynolds et al., 2013; Zhang Q. et al., 2018; Guiraldelli et al., 2018; Zhang et al., 2019).

Like the budding yeast *zip1Δ* mutant, knockout mutation of components of the SC central region, SYCP1, SYCE1, SYCE2, SYCE3, TEX12, and SIX6OS1, in mice abolishes both synapsis and crossing over (de Vries et al., 2005; Bolcun-Filas et al., 2007; Hamer et al., 2008; Bolcun-Filas et al., 2009; Schramm et al., 2011; Gomez et al., 2016). In *C. elegans*, mutation of components of the SC central region (SYP-1, SYP-2, SYP-3, and SYP-4) also causes a severe reduction or loss of crossovers (MacQueen et al., 2002; Colaiacovo et al., 2003; Smolikov et al., 2007a; Smolikov et al., 2007b; Smolikov et al., 2009), indicating a coupling between SC formation and crossing over in most organisms. A notable exception is *Arabidopsis thaliana,* in which meiocytes lacking the SC central element ZYP1 are defective for synapsis but form elevated numbers of crossovers (Capilla-Perez et al., 2021). Similarly, the absence of the central element proteins Ecm11 and Gmc2 in budding yeast causes defective SC formation but increased crossing over (Voelkel-Meiman et al., 2016; Lee et al., 2021). These observations suggest that full synapsis and the SC central region are not essential for crossing over *per se*, but may function to control a proper number of crossovers.

Despite the close link between SC formation and crossing over in most species, uncoupling of the two events is implicated in a meiosis-specific depletion mutant of a component of SCF (Skp1-Cullin-F box) E3 ubiquitin ligase, Cdc53. The budding yeast *cdc53* mutant is largely proficient in crossover formation, but is severely defective for the elongation of SCs and shows the abnormal accumulation of ZMM proteins (Zhu et al., 2021). Moreover, when Cdc53 depletion is combined with the *pch2* deletion mutation, lacking the AAA+ ATPase that removes Hop1^HORMAD1^ from synapsed axes, the formation of full-length SCs is restored, but now DSB repair and crossing over are stalled. This uncoupling is unexpected since most yeast mutants defective for meiotic DSB repair also impair SC elongation. A possible explanation is that SCF is part of a regulatory surveillance mechanism that couples SC elongation and DSB repair in meiotic cells.

## Crossover patterning on synaptonemal complexes

Crossovers, in concert with cohesion between sister chromatids, create connections between homologs called chiasmata that enable stable bipolar orientation of homologs on the meiosis-I spindle and consequently accurate disjunction at the first meiotic division. The number and position of crossovers, and thus chiasmata, are strictly controlled: each pair of homologous chromosomes (a bivalent) obtains at least one crossover (the obligate crossover or crossover assurance) and when multiple crossovers form between a bivalent they are evenly spaced (crossover interference). Crossover homeostasis can maintain crossover numbers at the expense of non-crossovers to buffer against variation in DSB numbers and inter-homolog bias (Martini et al., 2006; Cole et al., 2012; Lao et al., 2013). In addition, the phenomenon of crossover covariation describes the observation that within individual nuclei, crossover frequencies covary across different chromosomes, which may have adaptative advantages by balancing the cost-benefit ratio of crossing over (Wang S. et al., 2019). The precise mechanisms of these crossover control processes remain unresolved.

In budding yeast, crossover interference has been analyzed genetically by analyzing the segregation patterns of linked gene alleles and spore autonomous fluorescent makers in tetrads (Cao et al., 1990; Sym and Roeder, 1994; Shinohara et al., 2003; Thacker et al., 2011; Lao et al., 2013); and in prophase-I nuclei by analyzing the distribution of crossover-specific Zip2 and Zip3 immunostaining foci along SCs (Fung et al., 2004; Zhang et al., 2014b). Zip3 foci are evenly spaced, implying the establishment of interference patterning at or before the time of Zip3 loading, which depends on DSB formation (Zhang et al., 2014b). Mutant analysis revealed that the SUMO-targeted ubiquitin ligase (STUbL), Slx5/8 and SUMOylation of Top2 and axis protein Red1 are required for crossover interference (Zhang et al., 2014c). These and other observations support the proposal of Kleckner and colleagues that crossover interference is mediated by the imposition and relief of mechanical stress along meiotic chromosome axes (the beam-film model; Kleckner et al., 2004; Zhang et al., 2014b).

ZHP-3 is a *C. elegans* RING-domain protein related to Zip3 and is essential for crossover formation (Jantsch et al., 2004). ZHP-3 functions with three paralogs (ZHP-1,2,4) inferred to act as two heterodimeric complexes ZHP-1/2 and ZHP-3/4 (Zhang L. et al., 2018). ZHP-3 localizes along SCs in two phases; first as multiple foci along each SC before becoming restricted to a single crossover-specific focus in late pachynema (Bhalla et al., 2008). In *C. elegans*, robust crossover assurance and absolute interference ensures that each pair of homologous chromosomes obtains exactly one crossover. *In vivo* imaging using Fluorescence Recovery After Photobleaching (FRAP) technology revealed the dynamic properties of the SC central region and a switch from a dynamic to a stable state as pachytene progresses, the timing of which coincides with crossover designation (Pattabiraman et al., 2017). Other *in vivo* imaging studies support the idea that the SC has liquid crystalline properties, suggesting that the diffusion of the ZHP complexes within the SC might govern crossover patterning *via* a diffusion-mediated or coarsening or condensation process (Rog et al., 2017; Stauffer et al., 2019; Zhang et al., 2021).

Diffusion-mediated coarsening as a mechanism for crossover patterning is also suggested from analysis in *Arabidopsis*. Both plants and *Sordaria* encode a sole RING-domain crossover factor called HEI10 (without Zip3^RNF212^ orthologs). The localization pattern of HEI10 is also dynamic: forming multiple discrete foci along the SCs in early pachynema, which then reduce in number until most foci have disappeared while a few sites accumulate HEI10 and mature into crossover sites marked by MutLγ (Chelysheva et al., 2012; Wang et al., 2012; De Muyt et al., 2014). Analysis of HEI10-focus patterning in several different contexts *via* super-resolution structure-illumination microscopy (SIM) imaging of fixed cells combined with modeling by computational simulation is compatible with diffusion-mediated coarsening of HEI10 foci as a mechanism for crossover patterning (Morgan et al., 2021).

**FIGURE 3:**
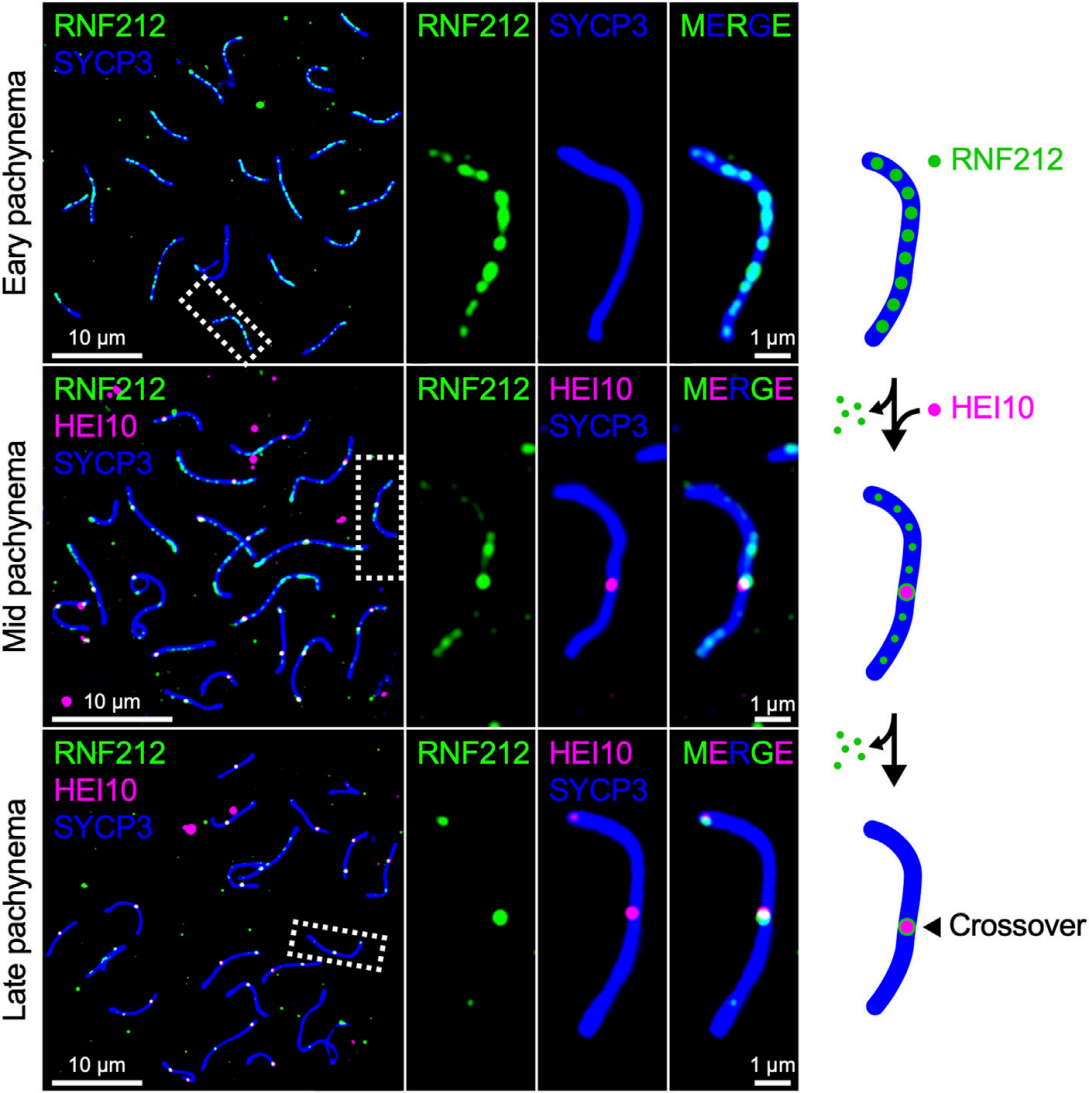
Chromosomal localization of RNF212 and HEI10 in mice. Successive stages of mouse pachytene spermatocytes immunostained for RNF212 (green), HEI10 (magenta), and SYCP3 (blue), HEI10 and SYCP3. RNF212 forms numerous discrete foci along the entire SCs (marked by SYCP3) in early pachynema before HEI10 foci emerge (top), loses most of foci but accumulates at HEI10-bound crossover sites in mid pachynema (middle), and eventually is restricted to crossover sites in late pachynema (bottom).

Mammals encode both Zip3 homolog RNF212 and HEI10, both of which are essential for crossover regulation in mice (Ward et al., 2007; Strong and Schimenti, 2010; Reynolds et al., 2013; Qiao et al., 2014). RNF212 shows dynamic localization along SCs similar to that of HEI10 in *Arabidopsis* and *Sordaria*, forming numerous discrete foci during early pachynema, which become restricted to crossover sites as pachytene progresses. By contrast, mouse HEI10 localizes only to crossover sites during mid-late pachynema and is not detected along SCs at earlier stages (Figure 3). It is suggested that RNF212-dependent SUMOylation stabilizes ZMM factors to confer crossover-competency to recombination sites, and HEI10-dependent ubiquitination subsequently licenses crossover/non-crossover differentiation by recruiting proteasomes to SCs to degrade as yet unknown factors (Rao et al., 2017). Importantly, the dosage of *Rnf212* and *Hei10* affects crossover rate in humans and mice, as seen for *Arabidopsis Hei10* (Kong et al., 2008; Chowdhury et al., 2009; Fledel-Alon et al., 2011; Kong et al., 2014; Ziolkowski et al., 2017). This similarity in the dosage effect on crossover numbers is consistent with the possibility that crossover patterning in mammals may also involve the diffusion-mediated accumulation of RNF212 and HEI10 at crossover sites.

## Discussion (perspective)

Meiotic chromosomes organize into specialized structures that help strictly regulate the number and position of meiotic DSBs, the choice of recombination template, and the differentiation of crossovers and non-crossovers to ultimately ensure the completion of DSB repair and accurate chromosome segregation. A diversity of approaches and model species are providing major insights into this molecular basis of the chromosome structure-recombination interface. However, major questions still remain to be addressed, including: Do cohesins and associated factors have direct functions in the regulation of meiotic recombination? Which factor(s) are responsible for TLAC formation in other organisms than yeasts, and how is TLAC formation coupled to DSB formation? How is inter-homolog bias established? What mechanisms underlie crossover patterning in mammals in which both Zip3/RNF212-family and HEI10-family RING-domain proteins are present? Recently, structural analysis of axis core proteins, Hop1/HORMADs, DSB proteins and associated proteins, and SC components is providing mechanistic insights into their functions (West et al., 2018; Boekhout et al., 2019; West et al., 2019; Sanchez-Saez et al., 2020; Claeys Bouuaert et al., 2021; Dunce et al., 2021; Rousova et al., 2021; Nore et al., 2022). Further mutant analysis based on protein structure will be a key to answer these unaddressed questions.

**FIGURE 4:**
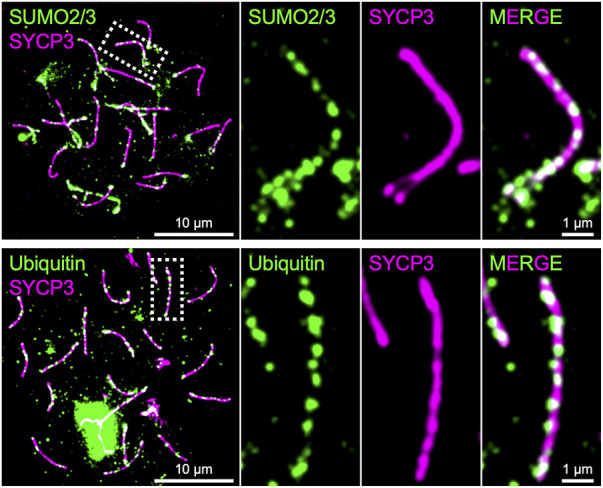
Chromosome axes decorated by SUMO and Ubiquitin in mice. Surface spreads of mouse spermatocyte chromosomes immunostained for SYCP3 (green) and SUMO2/3 (green; top) and Ubiquitin (green; bottom), respectively. Both SUMO and ubiquitin are enriched on chromosome axes where SYCP3 localizes.

As presented above, SUMO, ubiquitin, and proteasome are involved in the regulation of chromosome axis length and crossover interference in budding yeast, and presumptive SUMO and ubiquitin ligases, RNF212 and HEI10, are essential for crossover regulation in mice, highlighting central roles for the SUMO and ubiquitin-proteasome systems in meiotic chromosome organization and the regulation of meiotic recombination. Indeed, SUMO is enriched on chromosome axes and SCs in budding yeast, *Sordaria*, mice, and humans, and ubiquitin and proteasome have been localized to chromosome axes in budding yeast, *C. elegans*, and mice (Voelkel-Meiman et al., 2013; Klug et al., 2013; Brown et al., 2008; De Muyt et al., 2014; Ahuja et al., 2017; Rao et al., 2017; Figure 4). Numerous meiotic factors, including cohesin and recombination proteins, undergo SUMOylation in budding yeast (Bhagwat et al., 2021), and the SCF ubiquitin ligase, which regulates SC elongation in conjunction to Pch2^TRIP13^ in budding yeast (Zhu et al., 2021), localizes to synapsed chromosome axes and targets HORMAD1 in mouse (Guan et al., 2020; Guan et al., 2022). Future analysis will further elucidate molecular roles of SUMO and the ubiquitin-proteasome system in the regulation of meiotic recombination in conjunction with chromosome architecture.
